# Karyomorphometry on three polyploid species of *Arum* L. (Araceae, Aroideae)

**DOI:** 10.3897/CompCytogen.v8i1.6444

**Published:** 2014-03-11

**Authors:** Alessio Turco, Pietro Medagli, Antonella Albano, Saverio D’Emerico

**Affiliations:** 1 Dept. of Biological and Environmental Sciences and Technologies, University of the Salento, Via prov.le Lecce-Monteroni 6, Lecce, Italy; 2 Dept. of Plant Biology, University of Bari “Aldo Moro”, Via Orabona 4, Bari, Italy

**Keywords:** Allocyclic segments, karyotype asymmetry, karyotype evolution, *Arum apulum*, *Arum italicum*, *Arum maculatum*

## Abstract

In this study three polyploid *Arum* Linnaeus, 1753 species from Southern Italy were chromosomally investigated. *Arum italicum* Miller, 1768 was found to have 2n = 84 chromosomes and a karyotype composed of numerous asymmetric chromosomes. *Arum maculatum* Linnaeus, 1753 and *Arum apulum* (Carano) P. C. Boyce, 1993 were found to have 2n = 56 chromosomes. In the examined taxa some chromosome pairs were characterized by the presence of weakly coloured Feulgen-stained segments. The karyotype morphology of *Arum italicum* was found to be similar to that of *Arum maculatum*, but the more asymmetrical karyotype and numerous weakly coloured Feulgen-stained segments observed in the former suggest the existence of more extensive rearrangements. In contrast, *Arum apulum* was observed to have a symmetrical karyotype. The A_1_, A_2_ and SYi karyotype asymmetry indices are presented. The relationships between these taxa in terms of karyotype morphology and evolution are discussed.

## Introduction

The high biodiversity of *Araceae* Jussieu, 1789, with ca. 109 genera and over 3700 species (Mayo et al. 1997), reflects their ability to occupy a wide range of environments. This family also displays a large variety of life forms, from epiphytic to aquatic, attesting to extensive adaptive radiation during the Cretaceous period (Chase et al. 2006, Anderson and Janssen 2009). Some *Araceae* genera exhibit heat production (Minorsky 2003). Indeed, Lamark first noticed that the inflorescences of *Arum italicum* Miller, 1768, produced heat in 1778 (Meeuse 1973). It was subsequently shown that several *Araceae* taxa can produce heating up to 22°C above the environmental temperature (Meeuse 1959). This is related to the group’s biology, as heat increases the volatilization rate of its odour, facilitating pollination (Dafni 1984). Chromosome counts have been conducted for 862 *Araceae* taxa, with the number varying from 2n = 10 for *Typhonium jinpingense* Z. L. Wang, H. Li & F. H. Bian, 2002 to 2n = 168 for *Arisaema heterophyllum* Blume, 1835 and *Typhonium eliosurum* (Bentham) O. D. Evans, 1961 (Cusimano et al. 2012 and references therein).

In this study we conducted a karyomorphometric survey of *Arum* Linnaeus, 1753, a small herbaceous genus containing about 28 species (Lobin et al. 2007), five of which are found among Italian vascular flora (Abbate et al. 2005, Conti et al. 2007). *Arum maculatum* Linnaeus, 1753 and *Arum italicum* have rhizomatous tubers while *Arum apulum* (Carano) P. C. Boyce, 1993 has a discoid tuber (Bedalov and Küpfer 2005). Bedalov and Küpfer (2005) suggested that the discoid tuber shape may represent the ancestral state of *Arum* with respect to the rhizomatous form, and this was confirmed by molecular studies conducted by Espìndola et al. (2010).

From a karyological point of view, the basic number for the *Arum* genus is x = 14 (Petersen 1993) with most of the species diploid rather than polyploid (Prime 1980). *Arum maculatum* and *Arum apulum* are tetraploid (2n = 56), while *Arum italicum* is hexaploid (2n = 84) (Marchi 1971, Beuret 1971, Bedalov et al. 2002, Lendel et al. 2006, Bedini et al. 2012). Most of the polyploid *Arum* taxa have been reported to occupy broader geographic ranges than their diploid counterparts (Bedalov 1981). The distribution of *Arum italicum* extends from the Caucasus through the Mediterranean region to the Atlantic coast (Bonnier 1931, Meusel et al. 1965, Dihoru 1970, Bedalov 1975). According to Meusel et al. (1965), Terpò (1973) and Bedalov (1981), *Arum maculatum* is distributed across Central and Western Europe. The broader geographical range of *Arum italicum* and *Arum maculatum* with respect to diploids such as *Arum pictum* Linnaeus filius, 1782 or *Arum orientale* M. Bieberstein, 1808 (Prime 1980) may be therefore explained by their capacity to colonize new areas. However, the diploid *Arum alpinum* Schott & Kotschy, 1851 has a very wide distribution (Bedalov and Fischer 1995) and the tetraploid *Arum apulum* has a very limited distribution, restricted to Southern Italy (Puglia) (Carano 1934, Gori 1958, Bianco et al. 1994).

Cytological investigations of *Arum* chromosome numbers have sought to clarify its taxonomy (Gori 1958, Marchi et al. 1964, Beuret 1971, 1972, Marchi 1971, Bedalov 1975, 1981). D’Emerico et al. (1993) and Bianco et al. (1994) also described the karyotypes of six species for the genus, and found that the studied taxa all had a “basic karyotype” characterized by the presence of marker-chromosome pairs. Specifically, they noticed that the diploids’ 14^th^ pair is characterized by chromosomes with one satellite on the short arm and another on the long arm; this feature was also shown in pair 27 for *Arum maculatum* and *Arum apulum* (Bedalov et al. 1992, D’Emerico et al. 1993).

The purpose of this study is to acquire detailed new information on the karyomorphometry and chromosome structure of *Arum italicum*, *Arum maculatum*, and *Arum apulum* from Southern Italy.

## Materials and methods

Samples of *Arum italicum* were collected from various sites in Puglia and Lucania, while samples of *Arum maculatum* were collected near Muro Lucano - Potenza (Lucania) and *Arum apulum* near Quasano, Sammichele, Turi - Bari (Puglia) (Table 1). Only *Arum apulum* and *Arum italicum* are cultured in the Museo Orto Botanico di Bari (Bari). The nomenclature used for classification follows Boyce (1989).

**Table 1.** *Arum taxa* investigated and origin of samples.

Taxon	Locality	Collector
*Arum apulum*	Apulia: Quasano (Bari)	Medagli and D’Emerico 13.IV.2010
	Apulia: Sammichele (Bari)	Medagli and D’Emerico 15.IV.2010
	Apulia: Turi (Bari)	Medagli and D’Emerico 15.IV.2010
*Arum italicum*	Apulia: Quasano (Bari)	Medagli and D’Emerico 13.IV.2010
	Apulia: Sammichele (Bari)	Medagli and D’Emerico 15.IV.2010
	Apulia: Turi (Bari)	Medagli and D’Emerico 15.IV.2010
	Lucania: Matera	Medagli and D’Emerico 22.IV.2010
	Lucania: Grottole (Matera)	Medagli and D’Emerico 23.IV.2010
	Lucania: Pomarico (Matera)	Medagli and D’Emerico 23.IV.2010
*Arum maculatum*	Lucania: Muro Lucano (Potenza)	Medagli and D’Emerico 27.V.2010

Root-tips were pretreated in 0.3% aqueous colchicine at 20°C for two hours, and subsequently fixed for five min in a 5:1:1:1 (volume ratio) mixture of absolute ethanol, chloroform, glacial acetic acid and formalin. Hydrolysis was carried out at 20°C in 5.5 N HCl for 20 min (Battaglia 1957 a, b), then stained with Schiff’s reagent. Root tips were squashed in a drop of 45% acetic acid.

The nomenclature used for describing karyotype composition followed Levan et al. (1964). The karyotype parameters were composed following D’Emerico et al. (1996) and evaluated by calculating haploid complement lengths, the SYi index introduced by Greilhuber and Speta (1976) and the A_1_ and A_2_ indices proposed by Romero Zarco (1986). The SYi index describes the average symmetry of the karyotype, A_1_ is the intrachromosomal asymmetry index (i.e. the average position of the centromere in a karyotype) and A_2,_ is the interchromosomal asymmetry index (i.e. variation in chromosome length). As a standard procedure, chromosome metaphase plates from at least five cells were measured.

For Giemsa C-banding, a modification of Schwarzacher et al. (1980) was used, but unfortunately in these taxa C-Banding staining was unable to differentiate chromosomal or nuclear structures.

## Results and discussion

This study provides new cytological information on three polyploid *Arum* taxa. The present analysis is in agreement with the sectional segregation based on tuber structure in the classification of the *Arum* genus suggested by Boyce (1989).

In *Arum italicum* the chromosome number 2n = 84 (Fig. 2a) was observed in all the investigated populations, which is consistent with previous reports (Marchi 1971, Bedalov 1981). However, one individual from the Gargano Peninsula was found to have the chromosome number 2n = 85, as previously reported by Marchi (1971). The detailed karyotype morphology of this species consists of 38m+30sm+14st+2t chromosomes. Pairs 5, 7, 28, 33, 35 and 42 show weakly coloured segments with Feulgen-staining on the long arm, while pairs 9, 11 and 21 show these on the short arm, and pair 15 has a slightly Feulgen-stained segment on both arms. Pair 39 has a microsatellite on the short arm, while pairs 37 and 41 have a microsatellite on the short arm and a secondary constriction on the long arm (Figs 3, 4a).

**Figure 1.**
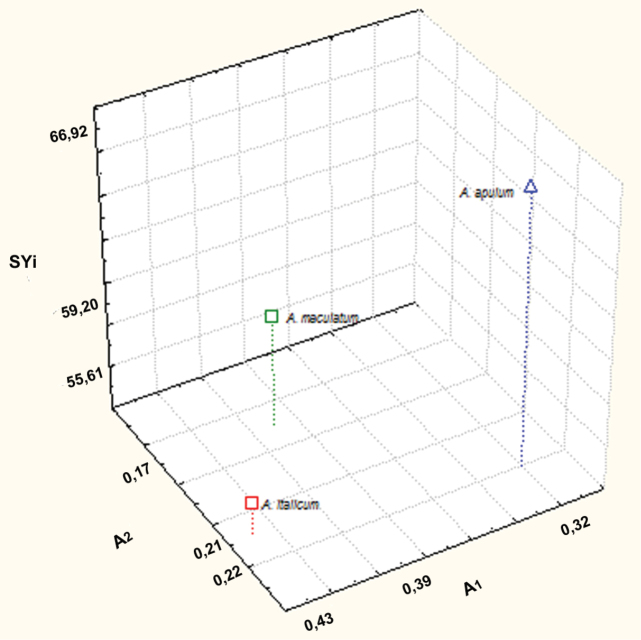
Scatter diagram of A1, A2 and SYi values of *Arum* taxa examined.

**Figure 2.**
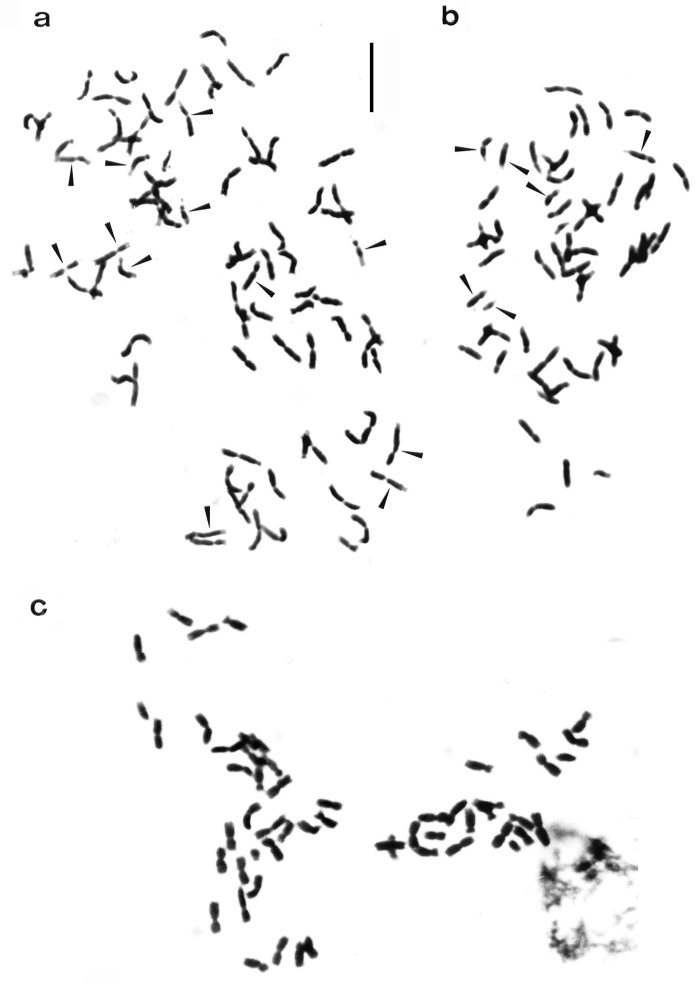
Somatic chromosomes of *Arum* species: **a** *Arum italicum* (2n = 84) **b** *Arum maculatum* (2n = 56) **c** *Arum apulum* (2n = 56). (Arrows show chromosomes with weakly coloured Feulgen-stained segments) Bar = 5µm.

**Figure 3.**
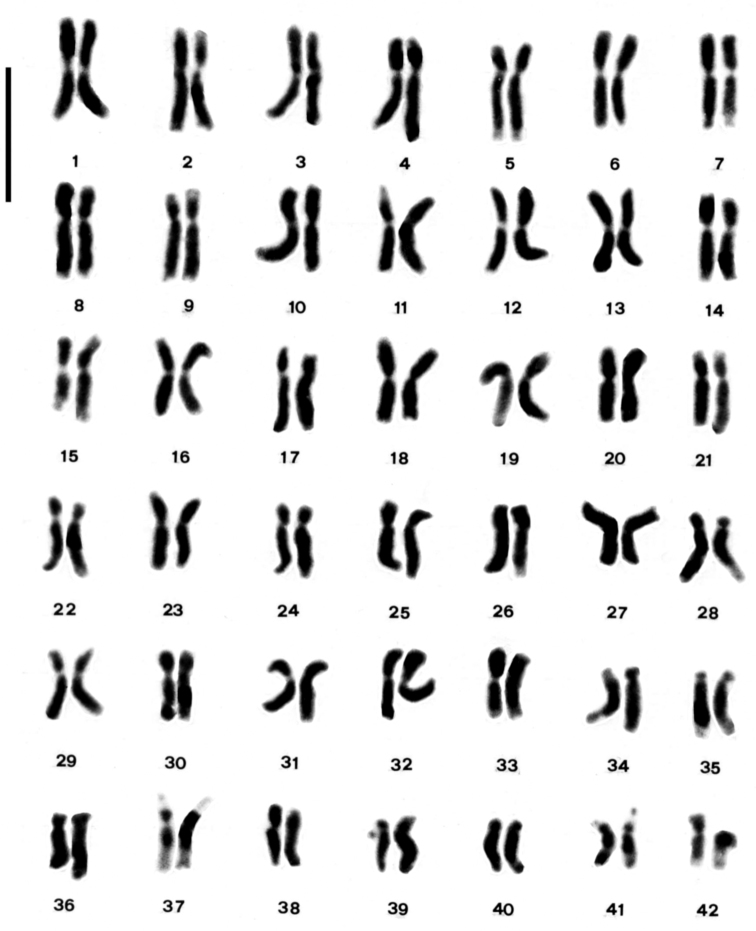
Karyotype of *Arum italicum*. Bar = 5µm.

**Figure 4.**
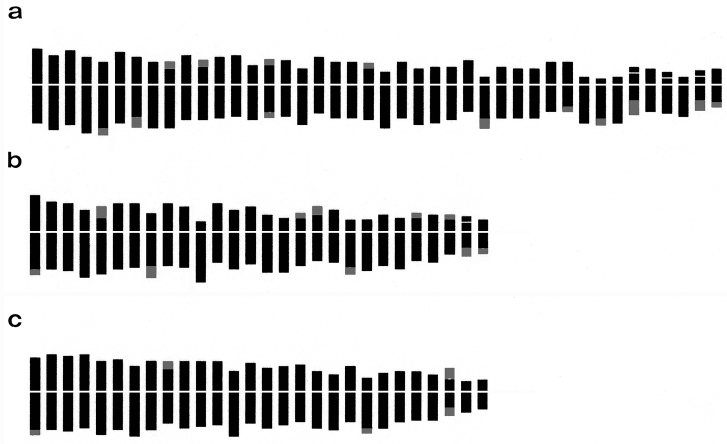
Haploid idiograms of *Arum* species: **a** *Arum italicum* **b** *Arum maculatum* **c** *Arum apulum*. (Telomeres shaded in gray show chromosomes with allocyclic segments).

*Arum maculatum* was found to have 2n = 56 somatic chromosomes (Fig. 2b), confirming earlier counts for this species on samples from the Balkan Peninsula (Bedalov 1981, D’Emerico et al. 1993). Our analyses show that the karyotype is similar to the previous reports and that it is characterized by the presence of 26m+24sm+6st chromosomes. However, individuals from central Puglia showed some differences in terms of the number and position of secondary constrictions. Pairs 1, 6, 19 and 28 have weakly coloured segments with Feulgen-staining on the long arm, while pairs 5, 18, 20 and 24 have these on the short arm and pair 27 has a microsatellite on the short arm and a secondary constriction on the long arm (Fig. 4b).

The samples of *Arum apulum* from Quasano, Sammichele and Turi (Bari) showed 2n = 56 chromosomes (Fig. 2c), in agreement with previous reports (Bianco et al. 1994). This species is characterized by a rather symmetrical karyotype, comprising mainly metacentric chromosomes. The karyotype morphology consists of 40m+16sm chromosomes. Pairs 1, 6 and 18 have weakly coloured segments with Feulgen-staining on the long arm; pair 16 has these on the short arm and pair 27 has a secondary constriction on the short arm and a microsatellite on the long arm (Fig. 4c).

The karyotype morphology of *Arum italicum* is similar to that of *Arum maculatum*. *Arum italicum* shows a more asymmetrical karyotype, with a higher intrachromosomal asymmetry index (A1 = 0.43) than *Arum maculatum* (A1 = 0.39). By contrast, *Arum apulum* possesses the most symmetrical karyotype of the three (A1 = 0.32) (Fig. 1, Table 2), being composed of mainly metacentric chromosomes and having few allocyclic segments. According to Stebbins (1971) the presence of metacentric chromosomes in the karyotype could be considered indicative of early divergence by a species. On the other hand, geographical isolation accompanied by ecological variation seems to support the current karyotype structure of *Arum apulum*.

**Table 2.** Morphometric parameters (mean ± S. E.) of the karyotypes of three *Arum* taxa studied. Haploid complement length; Chromosome number; A_1_, A_2_ (Romero Zarco 1986) and Syi (Greilhuber and Speta 1976).

Taxa	Haploid complement (µm)	Chromosome number 2n	A_1_	A_2_	SYi
*Arum apulum*	90.58 (± 3.12)	56	0.32 (± 0.01)	0.22 (± 0.01)	66.92 (± 1.61)
*Arum maculatum*	96.63 (± 2.46)	56	0.39 (± 0.01)	0.17 (± 0.01)	59.20 (± 0.27)
*Arum italicum*	169.22 (± 16.36)	84	0.43 (± 0.02)	0.21 (± 0.02)	55.61 (± 1.90)

In all the examined taxa some chromosome pairs are characterized by the presence of weakly stained segments, formerly described as secondary constrictions (D’Emerico et al. 1993). Dyer (1963) and Vosa and Colasante (1995) reported that similar segments have been found in several groups of plants (e.g. *Gasteria* Duval, 1809, *Iris* Linnaeus, 1753, *Aloe* Linnaeus, 1753). Moreover, they suggest that in somatic metaphase some chromosomes can exhibit non-contracted telomeric segments called “allocyclic segments”. Vosa and Bennett (1990) and Bennett and Grimshaw (1991) suggested that the presence of this type of segment could be used to distinguish species with similar karyotypes. In our study, *Arum italicum* showed numerous chromosomes with these segments, in contrast to *Arum maculatum* and *Arum apulum*. Polyploidy associated with structural changes in chromosomes is involved in bringing about further diversifications of karyotype morphology (Stebbins 1971). Therefore, on this basis we suggest *Arum italicum* is characterised by more rearrangement in its chromosome complement than the other two species.
